# Prothèse totale du genou sans resurfaçage de la rotule: à propos de 60 cas

**DOI:** 10.11604/pamj.2020.36.132.15861

**Published:** 2020-06-25

**Authors:** Seddik Benchekroun, Mohammed Lahsika, Hatim Abid, Mohammed El Idrissi, Abdelhalim El Ibrahimi, Abdelmajid El Mrini

**Affiliations:** 1 Département de Chirurgie Traumatologique et Orthopédique, CHU Hassan II, Fès, Maroc

**Keywords:** Prothèse totale du genou, resurfaçage de la rotule, cartilage patellaire, Total knee replacement, patella resurfacing, patellar cartilage

## Abstract

Lors de la mise en place d’une prothèse totale du genou (PTG), une des principales problématiques de la technique chirurgicale est le choix à faire entre la conservation de la patella ou son resurfaçage avec remplacement du cartilage patellaire par une prothèse. Cette problématique continue à faire l’objet d’une controverse au sein de la communauté orthopédique. Il n'y a pas de consensus clair sur la gestion optimale de la rotule pendant arthroplastie totale du genou (PTG). Ce travail est une étude rétrospective concernant 60 arthroplasties totales du genou sans resurfaçage de la rotule, implantées au Service de Chirurgie Traumatologique et Orthopédique (B) du CHU Hassan II de Fès, depuis janvier 2009 à décembre 2015. L’âge moyen de nos patients était de 58 ans avec des extrêmes allant de 20 ans à 80 ans. La prédominance féminine est nette avec 41 femmes soit (72%) et 16 hommes soit (28%). Cliniquement, nos malades se plaignaient de douleurs de type mécanique dans la majorité des cas et tous nos patients ont bénéficié d’un examen clinique et radiologique. Le score IKS a été utilisé pour évaluer l’état du genou avant et après l’intervention. L’acte opératoire a consisté en une arthroplastie totale du genou par prothèse totale du genou sans resurfaçage de la rotule. Les complications post-opératoires étaient marquées par 7 cas de douleur antérieur de genou, 2 cas d’infections cutanées superficielles traitées par une antibiothérapie adéquate et 3 cas de raideur. Aucun cas d’hématome ou de phlébite ou de sepsis n’a été signalé. Les résultats cliniques après un recul de 18 mois ont été satisfaisants. À la lumière de ces résultats, la conservation de la patella a permis d’obtenir des résultats très satisfaisants à moyen terme concernant la douleur et la fonction. De plus, les complications potentielles des prothèses patellaires ont été évitées. Une seule contrainte demeure concernant l’avenir de ces prothèses implantées sans resurfaçage est l’apparition ou parfois la persistance de la douleur antérieur du genou d’où la nécessité d’un resurfaçage secondaire.

## Abstract (en)

During total knee replacement, one of the main problems of surgeon is to choose between preservation of the patella or resurfacing with prosthetic replacement of patellar cartilage. This issue continues to be a subject of controversy within the orthopaedic community. There is no clear consensus on the optimal approach to the patella during total knee arthroplasty (TKA). We conducted a retrospective study of 60 patients undergoing total knee replacement without resurfacing of the patella. These procedures were performed in the Department of Orthopedics and Trauma-Surgery (B) at the University Hospital Hassan II, Fez, since January 2009 to December 2015. The average age of patients was 58 years, ranging between 20 years and 80 years. Female prevalence was clear (41 women, 72% and 16 men, 28%). Clinically, the majority of patients had mechanical pain and all patients underwent clinical and radiological examination. The IKS score was used to assess knee condition before and after surgery. The surgical procedure was based on total knee arthroplasty without patellar resurfacing. Post-operative complications included 7 cases of anterior knee pain, 2 cases of infection of superficial skin treated with appropriate antibiotic therapy and 3 cases of stiffness. No cases of hematoma, phlebitis or sepsis were reported. Clinical results after a follow-up period of 18 months were satisfactory. Given these results, the preservation of the patella allowed for very satisfactory middle term results regarding pain relief and restoration of function. In addition, potential complications of patella prosthesis were avoided. One limitation of these replacements without resurfacing is the occurrence, or sometimes the persistence, of anterior knee pain; hence the need for secondary resurfacing. Current data from the literature show that there is no consensus on the approach to the patella during total knee replacement. Finally, patella resurfacing is an approach that the surgeon should choose on the basis of current data. This said, the debate can continue focusing on a single issue: which approach should be used to the patella.

## Introduction

L’arthroplastie du genou est indiscutablement un des grands succès de la chirurgie orthopédique moderne qui a révolutionnée le traitement des pathologies articulaires dégénératives et inflammatoires. Ses objectifs visent alors de lutter contre la douleur, de corriger les défauts mécaniques, d’améliorer la fonction articulaire et la qualité de vie des patients. Lors de la mise en place d’une PTG, une des principales problématiques de la technique chirurgicale est le choix à faire entre la conservation de la patella ou son resurfaçage avec remplacement du cartilage patellaire par une prothèse. Cette problématique continue à faire l’objet d’une controverse au sein de la communauté orthopédique [1]. Il n'y a pas de consensus clair sur la gestion optimale de la rotule pendant arthroplastie totale du genou (PTG). L’objectif de notre travail est: analyser les résultats cliniques et radiologiques des patients traités par PTG sans resurfaçage; discuter l’indication du non resurfaçage de la rotule; évaluer les complications suite à la mise en place d’une PTG sans resurfaçage; comparer ces résultats avec la littérature.

## Méthodes

Ce travail est une étude rétrospective concernant 60 arthroplasties totales du genou sans resurfaçage de la rotule, implantées au Service de Chirurgie Traumatologique et Orthopédique (B) du CHU Hassan II de Fès, depuis janvier 2009 à décembre 2015. On a inclus les patients qui ont bénéficié d’une mise en place d’une PTG de première intention sans resurfaçage de la rotule. On a exclu les PTG avec resurfaçage, les PTG de deuxièmes intentions et les patients ayant subi une arthroplastie prothétique de genou secondaire. Tous les patients ont suivi la même procédure chirurgicale et postopératoire. L'évaluation était basée sur les renseignements et paramètres retenus à partir des dossiers médicales des patients et tous enregistrée sur des fiches d’exploitation préétablis.

## Résultats

**Figure 1:**
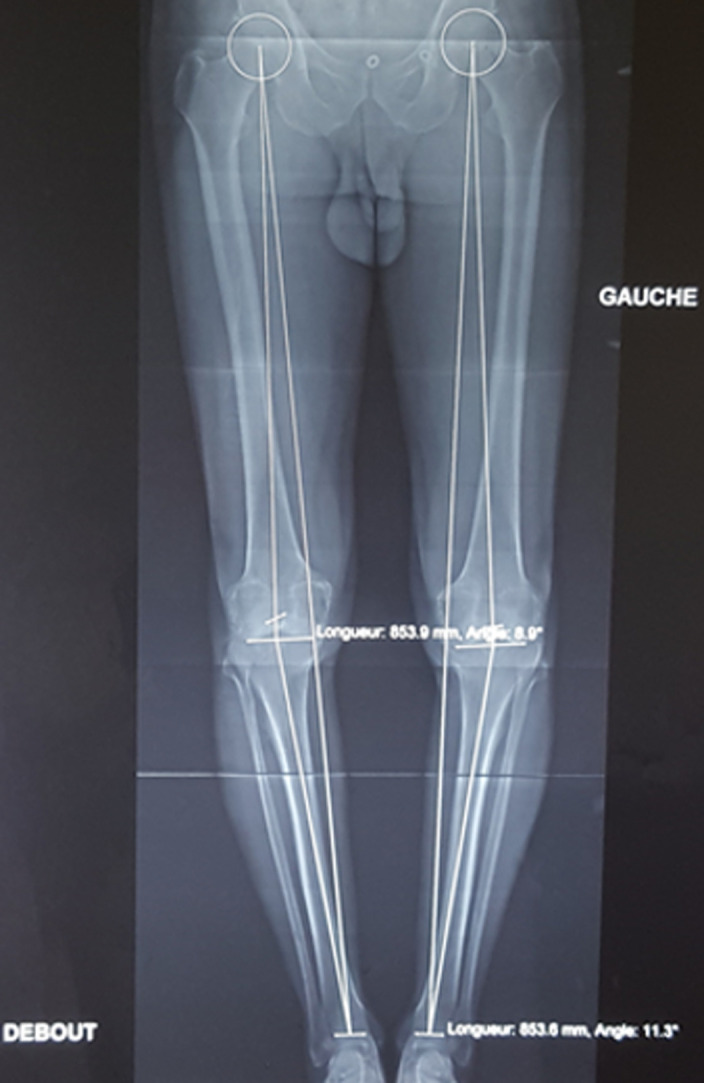
goniométrie montrant un genou varum bilatéral

**Figure 2:**
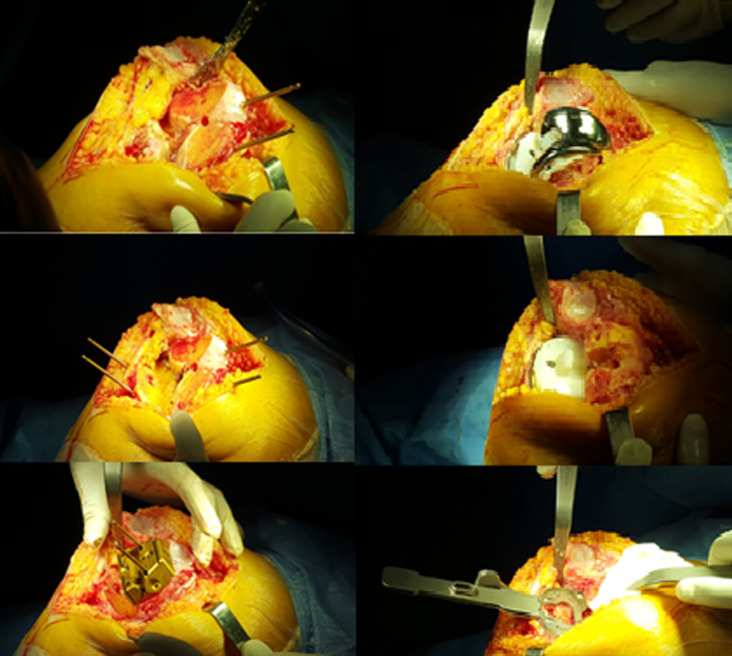
PTG en per-opératoire

**Figure 3:**
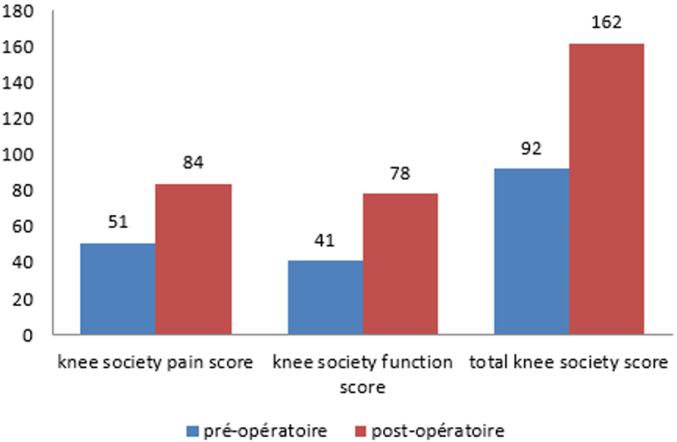
résultats du score IKS

L’âge de nos patients variait entre 35 et 84 ans, avec une moyenne: 58 ans. Soixante-dix-sept% de nos patients avaient un âge supérieur à 50 ans. La série comportait 57 patients avec une prédominance féminine dont 41 femmes soit (72%) et 16 hommes soit (28%). On note une prédominance féminine avec un sexe ratio de 0,4. Nous avons noté: 6 prothèses totales du genou bilatérales, soit 10%, 38 ont été posées à gauche (63%), 22 posées à droite (37%). L’arthrose était d’apparition primaire dans 85% de nos patients, par ailleurs l’arthrose était d’apparition secondaire chez 15% de cas répartis comme suit: 5 cas d’arthrites inflammatoires dans le cadre de polyarthrite rhumatoïde: un cas, l’arthrose est arrivée secondairement à une arthrite septique; un cas, l’arthrose s’est installée après des traumatismes (fractures de plateau tibial externe entrainant des varus conséquents et une rupture de LCA entrainant une instabilité) et un cas de tumeur maligne dans le cadre d’ostéosarcome. Soixante-cinq (65)% des PTG (37 cas) ont été réalisées chez des patients en surpoids ou obèses avec un IMC moyen des patients était de 26 kg/m^2^ [20-38] dont le poids moyen des patients était de 70 kg [57-110]. On note aussi que le 2/3 de nos malades consultent à un stade tardif, il s’agit soit de patients qui consultent pour la première fois, soit adressés tardivement par d’autres confrères principalement des médecins rhumatologues et des médecins généralistes. Le délai de consultation variait entre 03 mois et 3 ans. L’axe HKA (hip knee ankle) qui était en moyenne de 167,8° [174,7°-162°] (Figure 1). Dans notre série, nous avons pratiqué une voie d’abord antéro-interne trans- vaste -médial chez tous nos patients (Figure 2). La durée opératoire moyenne est estimée à 1h15. Toutes les prothèses réalisées dans notre série étaient cimentées. Nous avons évalué la douleur en se référant à l’échelle visuelle de la douleur en per et post-opératoire (Tableau 1). Nous avons opté pour la classification d’AHLBACK afin de classer les genoux arthrosiques selon leur stade radiologique: le stade II était présent chez 12 cas soit 20%, le stade III était présent chez 18 cas soit 30,5%, le stade IV était présent chez 27 cas soit 46%, le stade V était présent chez 2 cas soit 3,5%. En ce qui concerne l’usure fémoro-patellaire en préopératoire: on a procédé par la classification d’IWANO: stade I: 9%, stade II: 20%, stade III: 44%, stade IV: 27%. Nous avons eu recours au score IKS pour évaluer les résultats fonctionnels (International Knee Society) qui est largement utilisé à travers le monde, il mesure les paramètres classiques du genou: la douleur, la fonction et la mobilité articulaire. Le score IKS était en moyenne à 92 en préopératoire et à 162 en postopératoire (Figure 3).

**Tableau 1:** comparaison de la sévérité pré et postopératoire

	Douleur minime ( 0 à 4)	Douleur modérée (5 à 7)	Douleur sévère (8 à 10)
Préopératoire	7cas (12%)	18 cas (32%)	4 cas (7%)
Postopératoire	13 cas (23%)	10 cas (17,5%)	2 cas (3,5%)

**Figure 4:**
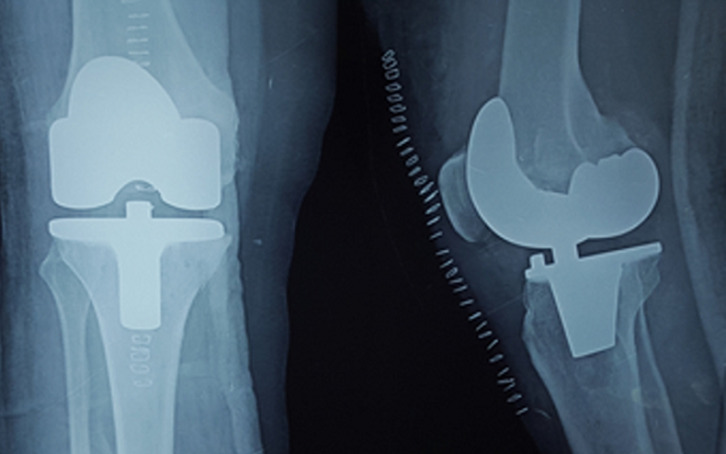
contrôle radiologique après une pose de PTG

**Complications:** nous avons trouvé des douleurs résiduelles postopératoires chez 7 cas, avec 1 cas d’infection superficielle jugulé par un simple parage et une antibiothérapie adaptée. On a signalé 2 cas de raideur avec une flexion à 60°. Tous nos patients ont été régulièrement suivis en consultation, ils sont revenus à la 3^e^ semaine puis 1 mois après, le 3^e^ mois puis chaque 6 mois. Le recul moyen était de 18 mois, avec des extrêmes de 6 mois à 5 ans (Figure 4). Quarante-deux genoux soit 70% avaient de très bon résultats c’étaient des genoux normo corrigés indolores, la flexion en postopératoire supérieure à 120°, périmètre de marche illimité sans boiterie ni utilisation de cannes.

## Discussion

Les partisans du non resurfaçage de la rotule argumentent leurs choix en se basant sur: [2] un cartilage viable avec un os non exposé à une anomalie ultérieure, une congruence fémoro-patellaire adéquate, un jeune âge du patient, une rotule de forme normale d'épaisseur appropriée, et aucun antécédent de cristallin ou synovite inflammatoire [3-5]. On note bien que parmi les indications les plus fortes pour défendre le non resurfaçage est une rotule très mince et sévèrement érodée où l'épaisseur de la rotule, même après résection conservatrice serait inférieure à 10-12 mm. Perspective internationale: actuellement, il y a 3 approches chirurgicales pour la prise en charge de la rotule au cours de l’arthroplastie totale du genou: toujours refaire surface; ne jamais refaire surface; sélectivement refaire surface en fonction des patients (qualité du cartilage articulaire et la congruence fémoro-patellaire au moment de la chirurgie). La grande variabilité dépend de l'emplacement et de la formation géographique: en Amérique du Nord, la majorité des chirurgiens ont tendance à refaire surface (>90%) car des essais multiples randomisés et méta-analyses bien faites ont montré un taux significativement plus faible de resurfaçage secondaire et éventuelle réintervention [6-10], une différence statistiquement significative de la douleur antérieure du genou, presque tous les essais ont montré que la douleur antérieure du genou est moins présente dans le groupe à qui ils ont refait surface, il est difficile de prédire la qualité ou l'épaisseur du cartilage articulaire rotulienne au moment de l'arthroplastie du genou, et combien de temps il va durer. En Europe, l'attitude envers la rotule est variable: refaire surface pour les mêmes raisons préconisées par les chirurgiens nord-américains. Ceux qui ne refont «jamais» de la surface de la rotule en opposition stricte car le taux de complications liées au resurfacage de la rotule est inacceptable. La philosophie de ces chirurgiens est de décider en peropératoire sur la base de la qualité du cartilage fémoro-patellaire. En Asie, la majorité des chirurgiens asiatiques ne refont pas surface à cause [2]: des patients avec de petites statures ceci dit une mince patella. Du coût supplémentaire impliqué au cours du resurfaçage. L'absence de registres nationaux établis et des essais randomisés bien conçus dans les pays asiatiques. Donc pour les chirurgiens, il est difficile de tirer des conclusions sur les données réelles de resurfaçage et du non resurfaçage. Les résultats fonctionnels ont été appréciés suivant le score IKS du genou (International Knee Society). Nos résultats encourageants rejoignent ceux de la littérature, la majorité des genoux étaient indolores ou peu douloureux, et ont récupéré une fonction satisfaisante (Tableau 2).

**Tableau 2:** répartition des patients selon leur score d´IKS

	Knee society pain score		Knee society function score		Total knee society score	
	Prép-op	Post-op	Pré-op	Post-op	Pré-op	Post-op
Seong hwan kim	37,6	91,5	42,9	86,5	81	178
Zhang tang liu	21,1	46	39,1	80,2	61	127
Hwang	53	96	44	79	97	175
Burnett	49	85	43	69	93	155
NOTRE SERIE	51	84	41	78	92	162

**Les complications:** il y a 2 principales complications du non resurfaçage de la rotule: [11-15] la douleur antérieure du genou et la nécessité pour un éventuel resurfaçage secondaire (Tableau 3). Le taux déclaré de resurfaçage secondaire sur la base de plusieurs études est de 10%-12% [16, 17].

**Tableau 3:** répartition des patients selon la douleur antérieure et le resurfaçage secondaire

Auteurs	Douleur antérieur du genou	Resurfaçage secondaire
Seong hwan kim	3	1
Zhang tang liu	8	1
Lauren beaupre	6	7
Burnett	10	4
NOTRE SERIE	7	1

## Conclusion

Dans notre étude, nous avons montré qu’en conservant la patella, nous avons pu obtenir des résultats très satisfaisants à court et à moyen terme en ce qui concerne l’amélioration de la douleur et de la fonction. De plus nous avons évité les complications potentielles liées à une prothèse patellaire. Une seule contrainte demeure concernant l’avenir de ces prothèses implantées sans resurfaçage est l’apparition ou parfois la persistance de la douleur antérieur du genou d’où la nécessité d’un resurfaçage secondaire. Les données actuelles de la littérature montrent qu’il n’existe pas de consensus sur la prise en charge de la rotule lors des prothèses totales de genou. Finalement, le resurfaçage de la rotule reste un choix qui doit être fait par le chirurgien sur la base des données actuelles, ceci dit que le débat continue.

### Etat des connaissances sur le sujet

- Une des principales problématiques de la technique chirurgicale lors d’une mise en place de PTG est le choix à faire entre la conservation de la patella ou son resurfaçage avec remplacement du cartilage patellaire par une prothèse.

### Contribution de notre étude à la connaissance

- La conservation de la patella a permis d’obtenir des résultats très satisfaisants à moyen terme concernant la douleur et la fonction;
- Les complications potentielles des prothèses patellaires ont été évitées;
- Le resurfaçage secondaire a été évitée chez tous nos malades.
